# Flame Retardancy of Biobased Composites—Research Development

**DOI:** 10.3390/ma13225253

**Published:** 2020-11-20

**Authors:** Anna Sienkiewicz, Piotr Czub

**Affiliations:** Department of Chemistry and Technology of Polymers, Cracow University of Technology, ul. Warszawska 24, 31-155 Cracow, Poland; pczub@pk.edu.pl

**Keywords:** bio-based polymers and composites, flame retardancy, natural fillers

## Abstract

Due to the thermal and fire sensitivity of polymer bio-composite materials, especially in the case of plant-based fillers applied for them, next to intensive research on the better mechanical performance of composites, it is extremely important to improve their reaction to fire. This is necessary due to the current widespread practical use of bio-based composites. The first part of this work relates to an overview of the most commonly used techniques and different approaches towards the increasing the fire resistance of petrochemical-based polymeric materials. The next few sections present commonly used methods of reducing the flammability of polymers and characterize the most frequently used compounds. It is highlighted that despite adverse health effects in animals and humans, some of mentioned fire retardants (such as halogenated organic derivatives e.g., hexabromocyclododecane, polybrominated diphenyl ether) are unfortunately also still in use, even for bio-composite materials. The most recent studies related to the development of the flame retardation of polymeric materials are then summarized. Particular attention is paid to the issue of flame retardation of bio-based polymer composites and the specifics of reducing the flammability of these materials. Strategies for retarding composites are discussed on examples of particular bio-polymers (such as: polylactide, polyhydroxyalkanoates or polyamide-11), as well as polymers obtained on the basis of natural raw materials (e.g., bio-based polyurethanes or bio-based epoxies). The advantages and disadvantages of these strategies, as well as the flame retardants used in them, are highlighted.

## 1. Introduction

Recently, great attention has been paid to different approaches to increase the fire resistance of bio-composites. This is an extremely important challenge to address, especially nowadays, when so much emphasis is placed on the industrial use of raw materials and materials of natural origin, preferably from renewable sources, including polymeric ones. Bio-based composites are already widely used, especially as construction materials for the automotive, aviation, and civil engineering, industries, as well as in areas such as electronics and electrical engineering, medicine, and astronautics. In these areas, next to the required mechanical strength or other usable parameters, their flammability is an important factor, sometimes even decisive for the possibility of using bio-based composites. 

In the literature, there are numerous definitions of bio-composites [1,2,3]. In general, a material is called ‘bio-composite’, if it is composed of two or more diverse components (one being naturally derived), which are combined to yield a new material with enhanced performance over individual constituent materials [4]. Commonly used classifications define bio-composites as composite materials in which at least one of the components is bio-based or biodegradable (although often the component or components are both bio-based and biodegradable). Usually, there are reinforced (generally fibre-reinforced) polymer composite materials with bio-based (generally also biodegradable) reinforcing agents and/or polymer matrix. The matrix in a such composite is made of polymers derived from preferably renewable or nonrenewable resources. On the other hand, the bio-based reinforcing agents are generally fibers from crops, crop processing by-products, and wastes. 

Bio-based composites already found numerous applications in diverse fields of our everyday lives, such as in the automotive industry (especially in car interior such as: seat backs, parcel shelves, boot linens, door linens, as well as door-trim panel and exterior auto body components [5,6]) or in building and construction, aerospace, and so forth. Sisal or bamboo fiber reinforced composites are applied, for instance, as permanent formwork, tanks, facades, long span roofing elements, and structural concrete elements [7,8,9].

Regardless of the obtaining method and origin of raw materials used for their synthesis, both petrochemical polymers and bio-polymers are organic compounds–hydrocarbons, and as such, they are highly flammable. The process of polymers burning is accompanied by the release of large heat energy and combustion products in the form of a suspension of fine particles of soot, ashes, and gas products in the air (smoke). The resulting fumes are generated in large quantities and generally are toxic. Also, crops are mostly flammable. However, due to the role of reinforcing agents, they are, in some sense, protected against heat and fire by a polymer matrix. In systems consisting of a bio-based polymer matrix and a bio-based reinforcing agent, polymer properties determine the thermal stability and fire resistance of such bio-composites. Therefore, it is expected that the polymers used as matrices are characterized by both high and long-term resistance to thermal degradation at temperatures up to 150–200 °C, shape stability during short-term heating to temperature about 400 °C, and low combustibility (as high as possible ignition temperature, time to ignition and limiting oxygen index, and as little as possible total heat release, heat release rate, volume of smoke production, and smoke production rate, as well as the amount of generated CO and CO_2_). Methods of reducing the flammability of polymers and polymeric materials obtained from petrochemical raw materials have been known for years but are constantly being developed [10]. The same methods are used for bio-composites [11,12]. Accordingly, in this paper, special attention is devoted to methods of reducing the flammability of composites in which the matrix is made of bio-polymers (produced by living organisms, biotechnological and chemical methods from raw materials of natural origin) and bio-polymer systems with the filler of natural origin in the form of plant fibers, as well as methods used for reducing the flammability of natural origin fibers. The presented manuscript not only gives an overview of the most commonly used techniques of different approaches towards the increase of the fire resistance of polymeric materials (including those obtained with the use of natural resources), but also describes the most recent studies related to the development of the flame retardation of bio-composite materials.

## 2. Strategies of Polymer Flame Retardancy

The thermal resistance and stability, as well as the flammability of polymers, is determined by several factors, including chemical structure (including, e.g., content of halogen, nitrogen or phosphorus atoms, type, number and position of functional groups), as well as physical structure—macroscopic and phase structure [13]. In the case of synthetic polymers, including those obtained from raw materials of natural origin, it is therefore possible to design their chemical composition and structure already at the synthesis stage in terms of the expected heat resistance and flammability. Similarly, it is possible in terms of their biodegradability. Hence, it is desirable to introduce into the structure of the polymer, especially in its main chain, cyclic, heterocyclic systems, aromatic rings, or a ladder structure, as well as amide, ester, carbonate, ether, disulfide, and sulphone groups [14]. The polymer should also be characterized by the highest possible value of glass transition temperature. 

The second way to reduce the flammability of polymers, very effective and widely used for many years, is the introduction of a non-flammable filler into the polymer, which reduces the content of the combustible component in the material—the polymer. In the role of fillers can be compounds that absorb heat delivered to the polymer during the smoking process and decompose endothermally, for example, by dehydration or decarboxylation (metal hydroxides and carbonates) [15]. The release of water molecules additionally delays the burning process of the material and reduces its temperature. An example of a such compound is hydrated aluminum oxide or aluminum hydroxide, which, by releasing water, decomposes into aluminum oxide, forming a protective layer on the polymer surface that effectively delays the spread of flame. In turn, magnesium hydroxide which acts similarly, is characterized by thermal stability greater than aluminum oxide (decomposition temperature in the range of 320–350 °C compared to 200–230 °C, respectively), greatly facilitating the processing of polymers with the addition of this filler.

The applied fillers are subjected to thermal degradation, forming a char, which cuts off the access of both heat and oxygen to the polymer. Boron compounds (zinc borate, ammonium borate, metal borates or borax) and silicon (silicas and organosilanes) [16] form an analogous barrier of boron or silicate glass. There are also fillers, introduced into the polymers, that under the influence of fire release gases acting as radical traps [17]. Also beneficial is the release of large amounts of non-flammable gases (e.g., N_2_, NH_3_, SO_2_ or CO_2_), causing the dilution of flammable and toxic gaseous combustion products, as well as oxygen, which reduces the burning rate and temperature. Moreover, fillers that increase the heat capacity of a material (e.g., antimony, zinc or iron oxides) work well. 

Another way to prevent the burning of polymeric materials is to protect their surfaces with coatings that are a natural barrier to oxygen and which under the influence of high temperature foam or melt [18,19]. In both cases, an insulating layer is created on the polymer surface to delay heat and mass transfer between phases. The introduction of a filler into the polymer is generally an effective and cheap solution due to the low cost of fillers. However, it should be remembered that it is possible to introduce only a certain amount of filler into the polymer without compromising its performance, primarily mechanical strength.

The most important way to delay smoking and reduce the flammability of polymers is to use compounds called retardants or flame retardants. The following compounds are suitable for this role: halogen, organophosphorus, halogen-phosphorus, as well as those containing elements such as antimony, boron, nitrogen, zinc, or tin. These compounds can be a physical additive to the polymer that remains chemically unconnected to the polymer matrix. The second group consists of reactive retardants, capable of being incorporated into the polymer structure through chemical bonds. This can be achieved by the introduction of a retardant at the stage of polymer synthesis (e.g., by copolymerization, polycondensation, or grafting) or by incorporating a flame retardant in the polymer matrix e.g., in the reactive processing or in the process of crosslinking).

## 3. Typical Flame Retardants for Polymer Materials

**Figure 1:**
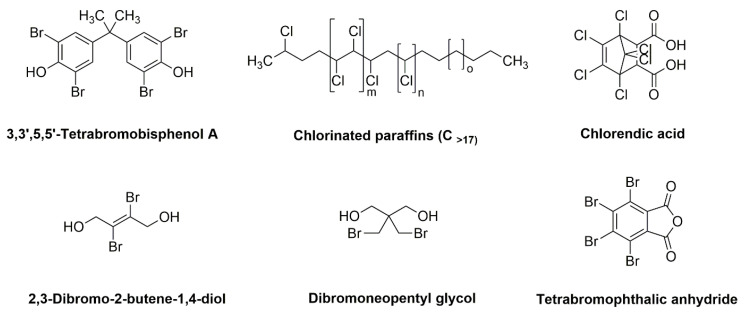
Examples of retardants based on halogen compounds.

Halogenated derivatives were used as the first retardants. Irrespective of their ability to form a barrier layer, they primarily generate halogen radicals (usually chlorine or bromine) contributing by recombination to terminate radical reactions occurring during combustion. The effectiveness of halogen derivatives as retardants increases in their group in a periodic table. However, mainly chlorinated or brominated organic derivatives (Table 1), aliphatic or aromatic hydrocarbons, selected for a specific polymer or suitable as substrates for polymer synthesis [20] exhibit practical importance (e.g., tetrabromobisphenol A in the synthesis of epoxy resins or chlorendic acid as a hardening agent for them, esters of chlorendic acid and glycols, as well as brominated diols and glycols as polyols in the synthesis of polyurethanes, or tetrabromophthalic anhydride in the synthesis of polyesters or long-chain chlorinated paraffins for thermoplastics, Figure 1).

In addition to the greater efficiency of bromine-containing compounds (which directly translates into a lower content of bromine in the retardant, necessary for an effective procedure), bromine-derived decay products are less volatile than chlorinated one, which is also a beneficial phenomenon. An interesting effect is the synergy of halogen compounds, e.g., with antimony trioxide, which ensures an effective action of a retardant with lower halogen content. In addition, antimony oxides effectively reduce the amount of smoke, which is generated during combustion.

**Figure 2:**
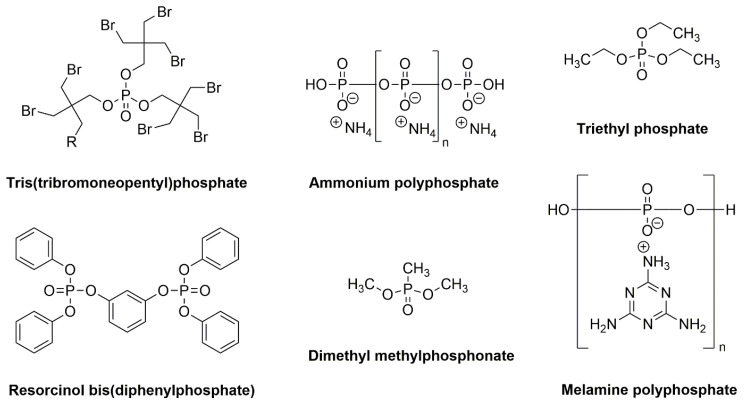
Examples of phosphorus-based retardants.

Currently, halogen derivatives are replaced with other flame retardants, due to the toxicity of their combustion products and the corrosive effect of HCl or HBr emitted during combustion. Many chemicals, which were which were commonly used as fire retardants only couple of years ago, are now recognized as global contaminants due to adverse health effects in animals and humans, including endocrine and thyroid disruption, immunotoxicity, reproductive toxicity, cancer, and adverse effects on fetal and child development and neurologic function [37,38,39,40]. Brominated flame retardants (e.g., mixtures of polybrominated diphenyl ethers or polychlorinated biphenyls) are a major source of toxic tetra- to octa-brominated dioxin and furan contamination. Moreover, brominated dioxins and furans are released during the entire life cycle of these flame retardants [41]. Inorganic and organic phosphorus compounds, e.g., ammonium polyphosphate, phosphate esters, triaryl phosphates, resorcinol bis(diphenylphosphate), aluminium diethyl phosphinate, and others, for example organo-nitrogen compounds—melamine polyphosphate or melamine bis(diphenylphosphate) (Figure 2), proved to be very effective retardants [42].

The effect of phosphate flame retardant is primarily to absorb heat in the melting process and to create a glassy barrier layer for oxygen and flame. Acid oxyphosphoric acid compounds, formed during the decomposition of phosphorus compounds, catalyze the dehydrogenation of polymers to carbon, facilitating the formation of char. Also, at the same concentration, phosphorus is a much more effective scavenger for radicals than chlorine or bromine [17]. In addition, compounds containing phosphorus and halogen or nitrogen atoms (e.g., melamine and guanidine and their derivatives) show a synergistic effect. Phosphorus compounds are used together with halogen derivatives with high halogen content. The disadvantage of flame retardants based on organophosphorus compounds is the need to use them in large quantities, which translates into a deterioration of the polymer properties.

An interesting group of compounds that can be used as flame retardants and introduced into the polymer in small amounts are nanomaterials: nanoclay (e.g., montmorillonite, sepiolite, and other layered materials), metal oxides, organic nanoparticles, carbon compounds (fullerenes [43,44,45], carbon tubes [46,47,48,49], and expandable graphite [50,51,52,53]) and silsesquioxane [54,55,56,57]. Aluminum silicates with nanotubes structure are also effective, e.g., halloysite [58,59,60] and imogolite [61,62]. Layered nanomaterials with a developed specific surface area, especially after their exfoliation in a polymer, constitute a barrier for both oxygen and gases released during combustion and contribute to the formation of an insulating layer of a char on the polymer surface. In turn, carbon compounds (most often used in the form of nanotubes, nanofibers and graphite [63]) are characterized by high thermal conductivity, due to which they increase the thermal conductivity of polymers, thus facilitating the removal of heat from the smoking zone and lowering the temperature of the material. 

It should be remembered that retardants, apart from their basic function—i.e., efficient and effective flame retardation—should be cheap and compatible with the polymer, not migrate from the polymer and remain in the polymer product during the entire period of use, should not impede polymer processing, not deteriorate its mechanical properties, and should not accelerate its aging. 

## 4. Flame Retardancy of Natural Fibers

The application of natural fibers as a reinforcement of polymer generates two main problems in the composite. The first one is the incompatibility of natural fibers as hydrophilic materials with mostly hydrophobic polymers. The second one is the flammability of natural fibers and the relatively low temperature of their degradation. In the case of cellulose-based fibers, their thermal decomposition proceeds according to various mechanisms, and begins with the release of moisture in the temperature range up to 100 °C. Further decomposition occurs in the temperature range of 200–340 °C, and lignin decomposition in the range of 160–400 °C [64]. In case of protein-based fibers, depending on their structure, thermal degradation begins even at 200 °C. The solution to both problems is chemical modification of fibers [65,66]. Here, it is worth nothing that higher cellulose contents in agricultural fibers enhance their mechanical properties, but induce higher flammability, in turn larger lignin contents result in greater char formulation hence retarding flame propagation [67]. Examples of natural fibers’ modification methods for their use as reinforcing fillers in bio-based composites are presented below.

**Figure 3:**
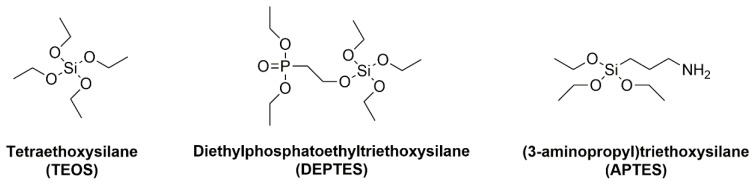
Chemical structure of silane precursors.

Flame retardancy can be provided by the reinforcement fibers or fabric, or by using a protective coating of fibers. Flax fabric used for the polyamide 11 composite application (Flax/PA11) can be treated with silane precursors, such as tetraethoxysilane (TEOS), diethylphosphatoethyltriethoxysilane (DEPTES), and (3-aminopropyl)triethoxysilane (APTES) (Figure 3) using two various sol-gel methods differing by the water content in order to increase the concentration of precursors in the media [68].

Various formulations of silane precursors alone and mixtures, with or without flame retardant additives in a form of aluminium phosphinate and mixture of aluminium phosphinate and melamine polyphosphate (AlPiMPP) were hydrolyzed using 0.1 M HCl in a mix of ethanol and deionized water. After immersing flax fabric samples for 1 h and 30 min in the prepared solutions, and drying process, the prepared reinforcement was applied using the method combining film stacking and vacuum bagging for the good impregnation of the fabric by the matrix. Based on the performed studies, it was found that depending on the used precursors and treatment method, the coatings homogeneously cover all along the fibers or aggregates. E.g., a smooth homogeneous coating is observed on the fibers in the case of TEOS-DEPTES treatment, whereas for TEOS, and TEOS-DEPTES-APTES treated samples, aggregates of various sizes are observed. The use of pure TEOS mainly leads to Q structures (bridging four oxygens per silicone—oxygen tetrahedron), whereas APTES or DEPTES leads to T structures (three oxygens attached). The fire-retardant properties of both the sol-gel treated fibers and fabrics, as well as the composites using such modified flax textiles are improved. The best results are obtained using a high concentration of silicon-containing precursors. The sol-gel coated fabric presents the decreased flame spread rate along the fabrics (exception—the multilayer treatment, leading to a rate similar to that of the untreated sample flame spread—about 1.3 mm/s). The increase in precursor concentration leads systematically to the decrease of the flame spread rate. It was found that, in general, fibers covered only by TEOS appears to be more efficient that the sol-gel coatings containing co-precursors (DEPTES, APTES, or DEPTES-APTES). Nevertheless, the best results were obtained with the sol-gel coatings containing the flame-retardant additives (0.61 mm/s for TEOS-AlPi compared to 1.3 mm/s for neat fiber). Additionally, due to the relatively low concentration of phosphorus in obtained sol-gel coatings, its presence does not sharply improve the flame retardancy of the flax/PA11 composites (depending on the applied ‘water-rich’/’water-reduced’ sol-gel procedure: 1.03/0.97 mm/s for TEOS versus 0.92/0.61 mm/s for TEOS-AlPi). The reduction of pHRR is observed for all composites containing sol-gel coated fibers. For the composites containing TEOS-treated fibers, the reduction of pHRR and THR is more visible with the higher concentration of sol-gel solution (char residue = 39 and 49 wt.%; THR = 43 and 29 MJ, respectively for ‘water-rich’ and ’water-reduced’ sol-gel procedure), as well as shortened time to ignition (33 and 28 s). 

**Figure 4:**
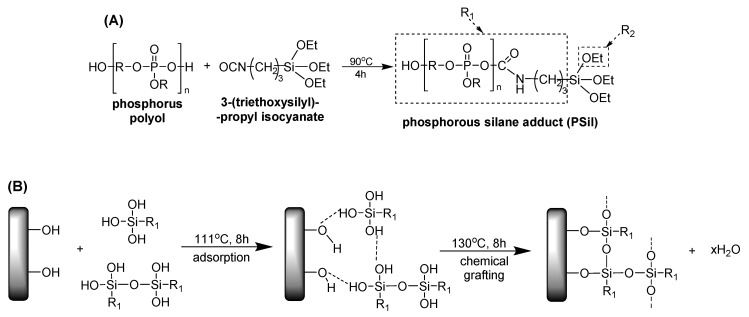
The synthesis of phosphorous silane adduct (**A**) and the treatment of flax fibers with PSil (**B**).

Phosphorous silane adduct (PSil), as a novel reactive modifying agent for cellulose-based bio-fibres, was synthesized by the reaction of phosphorus polyol and 3-(triethoxysilyl)-propyl isocyanate (Figure 4A). This toluenic mixture of flax fibres and PSil was refluxed at 111 °C for 8 h, then the obtained fibers were heated in an oven at 130 °C for 8 h in order to promote the hydrolysis of the alkoxy(triethoxysilane) groups of PSil to silanol moieties. The flax fiber treatment with PSil results in approximately 2 wt.% P content.

**Figure 5:**
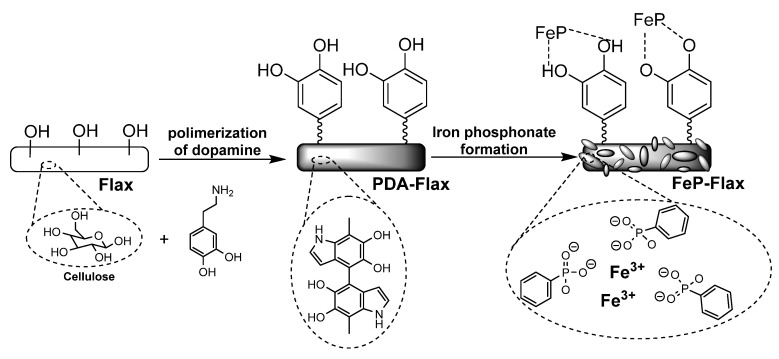
Surface modification of flax fiber.

Taking an advantage from studies on the properties of dopamine, a novel method of modification of natural fibers had been elaborated [69]. Dopamine in an aqueous solution exhibits an ability to spontaneously polymerize and form a thin polydopamine film on various types of surfaces, as well as the ability of catechol groups of obtained polydopamine film to form hydrogen bonds and metal-ligand complexes with various materials. In the presented solution, the surface of the raw flax fiber was modified by first coating with a thin adhesive polydopamine (PDA) film in an aqueous solution of dopamine, followed by the in situ growth of iron phosphonate on the fiber surface (Figure 5).

The obtained modified flax fiber is characterized by a LOI value of 26.1 and UL-94 V-2 rating. Moreover, it was found that bio-composite of PLA-modified flax fiber show high resistance to small flame ignition sources, and compared to material reinforced with raw flax fiber present a 16% and 21% decrease in peak of heat release rate (pHRR) and total smoke production (TSP), respectively. As a result of the catalytic effect of metallic compounds and phosphorus-containing acids, more oligomers are produced at the early thermal decomposition of bio-composite FeP-flax/PLA and the release of high flammable gaseous compounds, such as acetaldehyde or CO, is reduced. 

There are also numerous studies on the flame retardance behavior regarding other fibers (Table 2), such e.g., kenaf, hemp, wheat, sisal, bamboo, or alginate fibers [70,71,72].

In general, it was proven that the additional modification of fiber surface (including e.g., alkali, iron phosphate, or hybrid silane/alkaline pretreatment [80,81,82]), in most cases, increases the thermal stability of the developed fiber-reinforced bio-composites by preventing heat transfer and improving cross-linking density of the system fiber-polymer matrix, thereby strengthening the molecular structure of a composite.

More recently, very interesting studies related to increasing the fire resistance of bio-composites were performed using alginate fibers. Alginates, as anionic polymer have an ability to cross-link via the supramolecular interaction sites with most divalent metal ions [83]. Such a combination leads to the inherent flame retardancy of alginates by forming the stable char layers and significant reduction of combustive gases due to the metal-catalyzed graphitization of pyrolytic intermediates. It was found that all zinc alginate fibers are intrinsically flame retardant, with LOI values of over 27.0, as compared with about 24.5 for alginic acid fiber [84]. Moreover, the HRR and total heat release values of zinc alginate fibers (obtained from cone calorimeter) were significantly lower than those of alginic acid fibers. Additionally, it was found that with increasing zinc ion content both HRR and total heat release decrease. 

Another interesting application of alginate derivatives is the synthesis of novel flame-retardant composite material based on zinc alginate (ZnAlg) and nano-cuprous oxide (Cu_2_O) via an eco-friendly freeze-drying process and a sol-gel method [85]. The obtained composites exhibit high thermal stability and achieved nearly non-flammability (LOI of 58). Based on the obtained results it was found that incorporation of Cu_2_O and ZnAlg resulted in composite of improved flame-retardancy, compared to ZnAlg.

An interesting approach had been also introduced using a Layer by Layer (LbL) technique. The presented research employs deoxyribonucleic acid (DNA) and chitosan to synthesize eco-friendly coatings, which are characterized by promising flame-retardant properties [86,87,88]. It is worth nothing that due to the presence of precursor of phosphoricpolyphosphoric acid, deoxyribose—a polyhydric char source, and the nitrogen-containing bases able to release ammonia, DNA is considered to be an intrinsically intumescent compound. Using the LbL method cotton fabrics were immersed into the positively and the negatively charged solutions (5 wt.% aqueous solutions of chitosan and DNA) until 5, 10, and 20 bilayers were built. It was proven that the additional combination of DNA with chitosan, which has already been proven to be a promising carbon source when used in LbL coatings, promoted the formation of char which reduced the flammability of the obtained system (indicated by the increased limit oxygen index up to 24% and reducing the heat release rate by 40% during cone calorimetry tests). 

It is worth nothing that some studies indicate that natural fillers in bio-composites might be used to reduce the amount of commonly applied fire retardant additives, e.g., aluminum trihydrate (ATH) used in thermoplastic starch could be replaced by the coconut fiber (CF) from agricultural waste (10 phr of CF/30 phr of ATH, compared with 40 phr of ATH composite) [89]. As indicated, aluminum trihydrate decomposes endothermically into water and Al_2_O_3_, diluting fuel and forming an inorganic residual protection layer. By replacing a part of aluminum trihydrate with the coconut fiber, the thermal decomposition behavior is not significantly affected and a synergistic increase in inorganic-carbonaceous fire residue is observed. 

## 5. Other Methods of Reducing the Flammability of Bio-Based Composites 

### 5.1. PLA-Based Composites 

It was found that materials with improved flammability might be obtained through the introduction into the polymer matrix, at the stage of the synthesis of the composite, specific fillers, which act not only as the reinforcement, resulting in better mechanical properties of the obtained material, but also—which is equally important–as the specific flame retardants. Fillers applied for the synthesis of composites might either be characterized by their natural ability to act as flame retardant or are subjected to specific modifications towards, e.g., the introduction of the particular functional groups increasing the fire resistance of the final bio-composite. The most commonly applied as fire retardant in bio-composites are the phosphorous compounds [79,90]. There are also some studies on the incorporation of nanoparticles, such as expandable graphite [91,92], multi-walled carbon nanotubes [93] or sepiolite nanoclays [94].

**Figure 6:**
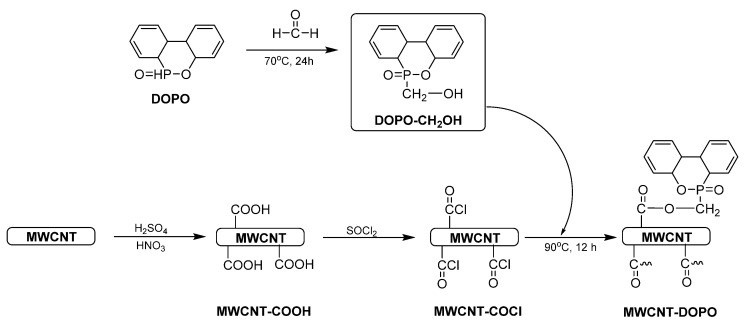
The synthesis of MWCNT-DOPO.

Carbon nanotubes (CNTs) have numerous applications due to their good mechanical, thermal, and electrical properties. However, their insolubility and tendency to aggregate makes their application problematic. An interesting solution is functionalizing CNTs with a 9,10-dihydro-9-oxa-10-phosphaphenanthrene-10-oxide (DOPO) to improve both flame retardancy and the dispersion of filler [95]. DOPO, which is characterized by excellent flame-retarding properties, is a cyclic phosphate with a diphenyl structure. It acts as flame inhibitor in the gas phase, or both in the gas and the condensed phase (by char formation) [96]. DOPO is grafted onto multi-walled carbon nanotubes (MWCNTs) by a three-step process (Figure 6).

DOPO covalently grafts on the surface of MWCNT and in that form functionalized MWCNTs (MWCNT-DOPOs) might be added as flame retardant into ramie/PLA composites (65 wt.% PLA and 35 wt.% MWCNT-DOPO). The performed studies indicate that novel fire retardant effectively increase char residue at high temperature and the char layer has a continuous structure, which protects the polymeric material. Due to the functionalization of MWCNTs with DOPO, carbon nanotubes are better dispersed in PLA and form a three-dimensional network in polymer matrix, which in higher temperatures acts as a framework supporting the produced carbonaceous chars. The char layer enhances the physical barrier effect, delaying the transfer of heat and volatile decomposition products out of the polymer. Furthermore, it could also act as a thermal shield for energy feedback from the flame. The addition of MWCNT-DOPOs also results in the improved tensile strength of ramie/PLA bio-composite. The introduction of unmodified MWCNT leads to the decrease of the tensile strength from 42.6 MPa to 41 MPa. However, with the application of MWCNT-DOPOs, probably due to the better dispersion of the filler, the tensile strength of the composite increases to 43.1 MPa. 

Expandable graphite, also applied for bio-composites, is included in a group of potential physical and inorganic intumescent flame retardant (IFR) systems, which are halogen-free, environmentally friendly, and highly effective [97,98]. An IFR system is usually composed of an acid source, a blowing agent, and a carbonic source. The performed studies indicate that the combination of expandable graphite and ammonium polyphosphate have satisfactory fire-resisting properties [99,100].

There are numerous studies on new fire retardants systems, however, for most of the recently published studies, one problem—developing a highly efficient fully bio-based flame-retardant composite, while maintaining or improving its mechanical properties—is common. Lately, based on the research on the various PLA composites, it was found that phytic acid (inositol hexaphosphoric acid), obtained from beans and cereal grains (such as corn, wheat, and sorghum), containing approximately 28% of phosphorous [101], has been point out as the one that exhibits large potential as a flame retardant for polymers with a minimal effect on their mechanical properties. For example, for the PLA bio-composites, it was found that the application of the combination of lignin and phytic acid is a simple route for developing bio-based flame-retardant systems. This approach combines two research findings: on one side, resulting from the aromatic structure EW lignin’s flame retardant properties—high thermal stability, and its ability of char forming [102,103], and on the other hand, enhanced flame retardant properties of lignin obtained by its chemical modification using phosphorus based compounds [104,105] or by its combination with phosphorus based flame retardant agents [106]. 

An interesting solution resulting from avoiding the lignin chemical modification and limiting its thermo-degradant effect during melt processing, involves the application of raw, untreated lignin and phytic acid in a PLA bio-based composite [107]. It was found that the incorporation of these two compounds alone in PLA have both positive and negative effects: the obtained materials are characterized by an improved fire behavior resulting from the formation of an insulating barrier of char during the combustion. Unfortunately, on the other hand, when using lignin alone, a significant PLA thermal degradation is observed, while the incorporation of only phytic acid increases the composite hygroscopic behavior. Surprisingly, it was found that the application of phytic acid/lignin blend into PLA limits the undesirable effects induced by each, in addition to significantly improving the fire behavior of PLA (while compared to plain PLA—the reduction of pHRR by 36 and 44%, for composite PLA/10 wt.% Phytic/10 wt.% Organosolv lignin and PLA/10 wt.% Phytic/10 wt.% Kraft lignin, respectively, and V-2 classification in UL-94 test). Moreover, it was found that the favorable interaction between the phytic acid and lignin results in a better dispersion of lignin in the composite and at the same time causes lignin’s functional groups to be less reactive, leading to decreased PLA thermal degradation. Additionally, for the composite PLA/15 wt.% Phytic/5 wt.% Organosolv lignin, it was observed that next to improved flame resistance (reduction of 36% of pHRR, compared to plain PLA) the application of the combination of phytic acid and lignin resulted in an increase of the elongation at break from 3.1% to 12.6%.

An another very interesting application of phytic acid is the synthesis of core-shell structured flame retardant, a bio-polyelectrolyte (PC), via the reaction of phytic acid with casein to microencapsulate ammonium polyphosphate (APP) [108]. The technique of microencapsulation is used here in order to provide a solution to overcome the incompatibility between the inorganic interface of APP and organic interface of polymer matrix. The invention describes the application of casein, benefiting from the presence of organic structures, P/N elements, the well dispersion within PLA matrix and the ability to form polyelectrolytes with other negative charged acids (such phytic acid) due to the high content of amino groups. It was found that the phosphate groups from phytic acid and the hydroxyl groups from casein can crosslink with APP and the formation of consolidate char layers enhance the thermal stability of PLA-composite. It was also found that the introduction of APP thought microencapsulation, as it enhances the interactions between flame retardant and polymer matrix, additionally improves the elongation at break and impact strength of the composite (e.g., from 7.0% of neat PLA sample to 11.5% of PLA/3.3% APP/1.7% PC). 

Taking an advantage of remarkable P/C atomic ratio (1/1) with the unique chelating ability of phytic acid with most metals [109] novel flame retardants based on system metal phytate were proposed. Based on studies on PLA metallic phytate composites containing 20 or 30 wt.% of bio-sourced phosphorous flame retardant additives, it was stated that the co-addition of aluminum phytate and sodium phytate is very promising approach, allowing for reaching a pHRR reduction similar to that obtained with 20 wt.% of Al-Phyt, while limiting the PLA chain degradation. This synergistic effect was also observed in the presence of either iron phytate or lanthanum phytate.

**Figure 7:**
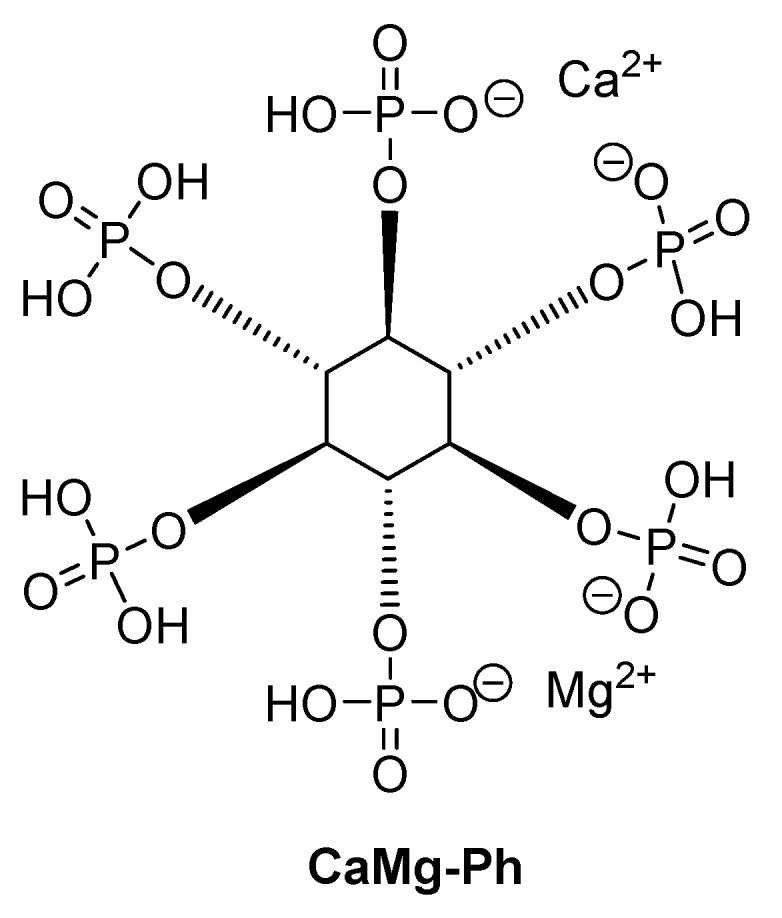
The chemical structure of CaMg-Ph.

Another example of such bio-flame retardant is calcium magnesium phytate (CaMg-Ph, Figure 7), synthesized via the reaction of calcium chloride and magnesium chloride with phytic acid. 

CaMg-Ph, as a bio-sourced phosphorous additive combined with acid-treated carbon nanotubes was tested as an fire retardant/mechanical reinforcement for the poly(lactic acid). It was found that the addition of 10, 20, and 30 wt.% of CaMg-Ph significantly reduced pHRR by 22%, 33%, and 38%, respectively, with a similar trend observed in total heat release. On one side, the addition of CaMg-Ph leads to the early degradation of PLA matrix and forming additional char residues, causing a supplementary barrier effect resulting in the reduction of pHRR and THR, while CNTs improve thermal stability and act as an effective reinforcement for CaMg-Ph flame retardant PLA systems. A PLA composite containing 19 wt.% of CaMg-Ph and 1 wt.% of CNT shows lower pHRR (35%) and higher char yield (18.4 wt.%) compared to PLA/CaMg-Ph20. Furthermore, the addition of the combination of 19 wt.% CaMg-Ph and 1 wt.% CNTs into PLA leads to a slight increase in tensile strength (52.8 MPa), higher than PLA/CaMg-Ph20 (50.4 MPa), which can be explained by the crystallinity recovery caused by the introduction of CNTs to the CaMg-Ph flame retardant PLA system and strengthening the bio-composite. 

**Figure 8:**
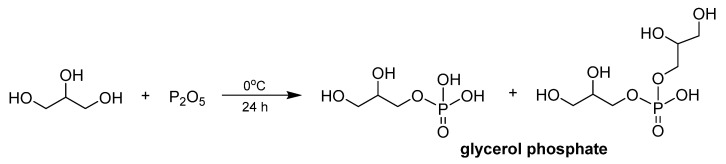
The synthesis of glycerol phosphate.

For the bio-composite polylactic acid-thermoplastic starch (80 wt.% PLA/20 wt.% TPS) an interesting solution involving replacement of glycerol, which is the most commonly used plasticizer of starch, was introduced [110]. In the performed research, glycerol was substituted by glycerol phosphate (GP), synthesized through the green, addition-type reaction between glycerol and phosphorus pentoxide (Figure 8).

Additionally, PLA/TPS bio-based composites could be filled with flax fibers modified through the one-step reactive flame-retardant treatment (see Figure 4). The use of the glycerol phosphate plasticizer resulted in a significant improvement of flame retardancy of TPS. The approximately 1 wt.% of P introduced through the plasticizer into the TPS-GP resulted in V-1 rating, high LOI value and 44% reduction of the total heat release. The application of modified starch, when applied in PLA, enhances char promoting capability, and thus provides improved flame retardancy to PLA/TPS blends. It was found that the combined application of the PSil-surface treated flax fibers and plasticizer in bio-composites resulted in flame retarded material with well-balanced strength and stiffness (30% reduction of peak of heat release rate compared to the phosphorus-free reference bio-composite). Furthermore, due to the beneficial effect of the presented multifunctional additive system, a loading of as low as 10 wt.% of ammonium polyphosphate (APP) could additionally decrease LOI by 33% and reduce heat emission by 23% (tHR of 57.4 MJ/m^2^), compared to 24% and 81.0 MJ/m^2^, respectively for PLA/TPS-GP/flax-PSil). 

Recently, an another interesting approach to the problem of improving both the flame retardancy and the mechanical properties of bio-composites based on the PLA was introduced. This study [111] implemented the Layer-by-Layer (LbL) method for the simultaneous improvement of the flame retardancy and the mechanical properties of PLA bio-composites. The LbL technique was used to modify the surface of flax fabrics using a quad-layer architecture encompassing chitosan, sepiolite, and ammonium polyphosphate. The deposition of 2.5 QL led to the formation of a homogenous coating leading to the significantly improved flame retardancy and fire safety of the prepared composites (LOI = 25.3%, reduced flame spread rates and the substantial reduction in peak of heat release rate—33% and maximum average rate of heat emission—30%), as well as improved mechanical properties (improved modulus and limited reductions in flexural strength).

### 5.2. PHA-Based Composites 

**Figure 9:**
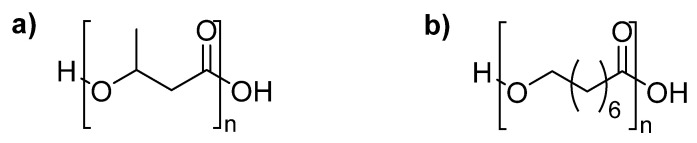
Chemical structure of polyhydroxyalkanoates: (**a**) poly-3-hydroxybutyrate (P3HB); and (**b**) polyhyxyoctanoate (PHO).

Like PLA, polyhydroxyalkanoates (PHAs, Figure 9) are polyesters derived from bio-based resources: PLA—from plants, but PHAs are produced and secreted out by bacteria.

**Figure 10:**
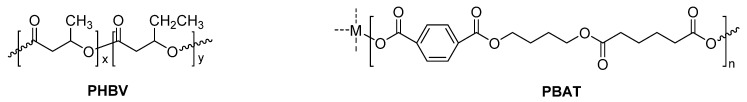
Chemical structure of PHBV and PBAT.

As for other bio-composites, in order to preserve the ecological character of PHAs, it is very important to avoid toxic halogen-based flame retardant for the benefit of the halogen-free fire retardant systems, such as metal oxides and phosphorous derivatives, e.g., phosphinates. Recently, according to the mentioned environmentally friendly approach, in the blend of poly(3-hydroxybutyrate-co-3-hydroxyvalerate)/poly(butylene adipate-co-terephthalate) (PHBV/PBAT, Figure 10), in order to improve flame retardancy and obtain materials characterized by satisfactory thermo-mechanical properties, the combination of aluminium phosphinate (AlPi), nanometric iron oxide and antimony oxide has been introduced [112]. 

**Figure 11:**
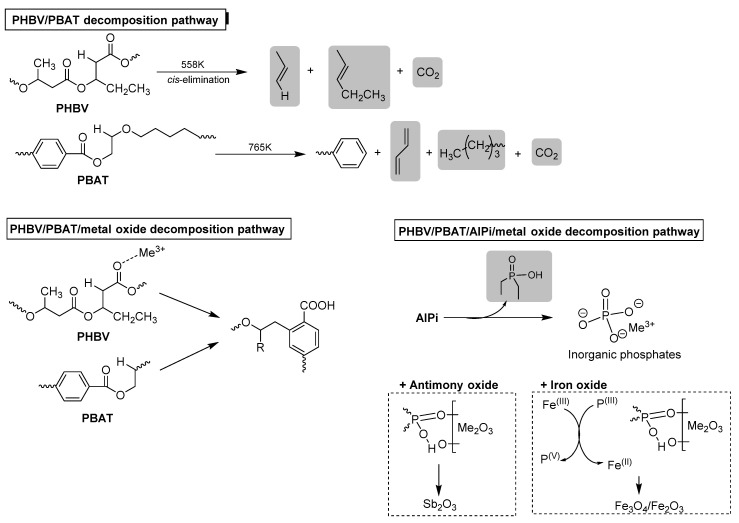
Schematic illustration of decomposition of neat blend PHBV/PBAT blends containing metal oxide (PHBV/PBAT/metal oxide) and both AlPi and metal oxides (PHBV/PBAT/AlPi/metal oxide). Products identified in the gas phase are highlighted in grey frames.

Poly(butylene adipate-co-terephthalate) is an aliphatic-aromatic copolyester of butylene adipate and terephthalate, that degrades naturally within few weeks throughout the enzymatic degradation and it is used in order to improve the elongation and toughness of bio-blends with a simultaneous reduction in tensile strength and modulus compared to the neat matrix [113]. Blend PHBV/PBAT (of the weight ratio 30:70) containing AlPi (8 wt.%), and metal oxides (2 wt.%), is prepared by melt blending. Based on the performed studies, it was found that both aluminium phosphinate and the metal oxide nanofiller simultaneously participate in the flame-retardancy mechanism. While AlPi acts as flame inhibition in the gas phase, the metal oxide stabilizes intermediate structures containing oxygen, promoting cross-linking in the solid phase between polymer chains and interacting with phosphorus-based intermediates. The improved fire retardancy of obtained blends is identified as an increase in intermediate char (8.4 wt.% for PHBV/PBAT/AlPi/Sb_2_O_3_ and 9.7 wt.% for PHBV/PBAT/AlPi/Fe_2_O_3_ compared to 2.3 wt.% for neat PHBV/PBAT, 2.8 wt.% for PHBV/PBAT/AlPi and 3.7 and 3.1 wt.% PHBV/PBAT/AlPi/Sb_2_O_3_ and PHBV/PBAT/AlPi/Fe_2_O_3_, respectively) and favored improvements in the UL 94 classification (V-0 and V-2, respectively for PHBV/PBAT/AlPi/Sb_2_O_3_ and PHBV/PBAT/AlPi/Fe_2_O_3_ compared to HB for PHBV/PBAT). It was found that while Fe_2_O_3_ reacts with the phosphinate additive in a redox cycle (the iron is reduced to Fe_3_O_4_ and the phosphorus is oxidized to inorganic phosphates), Sb_2_O_3_, as non-reducible oxide, might have a catalytic effect on the cross-linking process (Figure 11).

Another interesting solution combining relatively good mechanical properties with fire retardancy performance in bio-polymer composites is obtaining a bi-layer laminate system based on biodegradable polyhydroxyalkanoates [114]. Two layers can be prepared using PHBV/PBAT, where a first layer characterized by flame-retarded properties is obtained by combining 8 wt.% aluminium diethylphosphinate flame retardant and 2 wt.% nanosized antimony oxide and the second fiber-reinforced layer contains 30 wt.% of kenaf fibers. Used fibers, besides providing better flexural and impact properties, promote the formation of a stable layer during combustion, which prevents heat and flammable volatiles from penetrating into the flame zone, resulting in similar or better resistance to fire of laminate structures compared to fully retarded compositions. Based on the performed studies, it was found that hydroxyl-rich kenaf fibers act as a carbonization agent, creating numerous holes and cavities, which affect the release of pyrolysis gases.

### 5.3. Other Bio-Based Polymer Composites

Polyamide-11 (PA11) is a bio-based polymer synthesized using 11-aminoundecanoic acid from castor oil [115]. It is a versatile thermoplastic polymer that exhibits excellent mechanical and chemical properties. However, pure PA11 is characterized by high flammability and gives rise to extended dripping during combustion. The improvement of the fire resistance of PA11-based materials might be performed by the addition of nanoparticles flame retardants (e.g., montmorillonite clay [112,113,116,117] and carbon nanofiller/nanotube with phosphorus flame retardants) during melt compounding process [118].

Zinc phosphinate (ZnP) and low sulphonate content alkali lignin (LS) might be used as fire retardants for polyamide 11 [119]. In the studied compositions, prepared by melt extrusion, the total loading of flame retardant and carbon source was kept on the level of 20 wt.%, where ZnP, as the major component, was varied from 10 to 15 wt.% and lignin from 5 to 10 wt.%. It was found that LS alone, due to the low T_max_ that favors the combustion process, is not remarkably effective as a flame retardant of PA11. The best fire behavior was achieved for the composition containing 10 wt.% of both additives. It was found that, due to the formation of a stable char layer, the interactions between LS and ZnP enhance flame-retardant properties, resulting in the reduction of pHRR (by about 50%), THR (about 13%) and maximum value of the average rate of heat emission (by 35%).

Both additive and reactive flame retardants are used to prepare fire resistant polyurethane materials. Additive flame retardants are suspended in the polymer matrix during the preparation. However, they have a possibility to migrate, compromising fire retardancy over time. On the other side, the reactive flame retardants create rigid bonds with polymer structure. The flame retardancy of PU-based composites has been thoroughly studied. Relatively large group of applied compounds—very effective, but toxic and giving dense smoke—constitute halogen-containing flame retardants [120]. On the other side there are intumescent flame retardants [121] and phosphorus-containing flame retardants [122], which are quite effective in enhancing the flame retardancy without producing toxic smoke.

**Figure 12:**
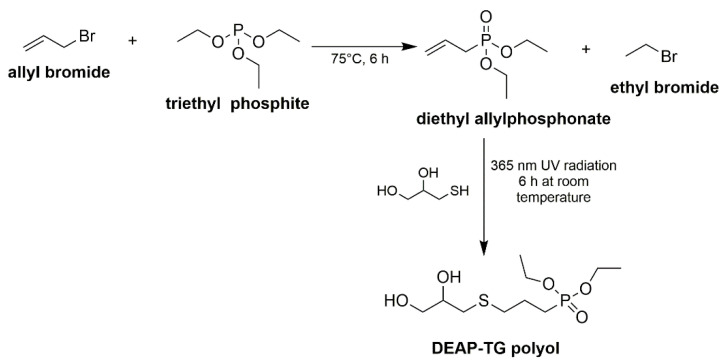
The synthesis of DEAP-TG polyol.

The enhancement of flame retardancy of polymer matrix is an important stage in the process of the synthesis of bio-composites with improved fire resistance. An interesting solution is the introduction of a fire retardant within polyol used for the synthesis of PU. Such a polyol might be obtained using diethyl allyl phosphonate (DEAP) and thioglycerol (TG) and applied along with different bio-based polyols derived from e.g., soybean oil, orange peel oil and castor oil [123]. DEAP-TG polyol is synthesized in two steps (Figure 12).

This procedure of the synthesis of bio-based PU might be an important stage for developing an effective method of production of PU bio-based composites with enhanced flame retardancy. It was found that by the addition of DEAP-TG polyol, the self-extinguishing time of soybean-based PU foam drops down from 157 s to 20.3 s and further to 3.1 s, respectively for the content of 0 wt.% P, 0.77 wt.% P and 1.5 wt.% P. Moreover, based on obtained results, the highest weight loss is observed for orange oil-based polyurethanes (reduction from 61% to 15% for 0.77 wt.% P and further to 5.2%, for 1.5 wt.% P). Furthermore, the formation of a protective char layer over the surface of the PU material due to decomposition of a phosphorus compound improves flame retardancy; materials containing 1.5 wt.% P, compared to those without phosphorous, exhibit a significant reduction in peak heat release rate, total heat release, total smoke release, and overall smoke production rate for polyol. 

At the same time, it is worth mentioning here that there are numerous studies on the improvement of flame retardancy of polyurethane-based bio-composites using expandable graphite [124,125,126]. For example, expandable graphite might be an effective flame retardant for rigid polyurethane and polyisocyanurate (PUR-PIR) foams based on bio-polyols [127]. In obtained PUR-PIR materials 70 wt.% of the petrochemical polyol is replaced by the rapeseed oil-based polyols obtained using two methods: the epoxidation with opening oxirane rings and transesterification with triethanolamine. The polyol premix consists of petrochemical polyol, a rapeseed oil-based polyol, the catalysts, the surfactant, water, and expandable graphite in an amount of 3, 6 or 9 wt.% per total foam mass. It was found that with the increased content of expandable graphite in polyurethane-polyisocyanurate system, the obtained composite exhibits better thermal properties and flame resistance properties due to the forming a stable char layer on the surface of foams (oxygen index of 26–27% and the peak of the heat release of 120–130 kW/m^2^).

Among numerous solutions introduced for PU-based bio-composite, the research work on potential synergistic effect between organically modified nanoclay and flame retardants on improving the flame retardancy and fire behaviour of rigid polyisocyanurate-polyurethane (PIR) foams nanocomposites [128], seems very interesting. PIR foam nanocomposites have been successfully prepared by *in-situ* polymerization, using renewable rosin polyester polyol. The application of such bio-polyol on one side results in higher thermal and dimensional stability than conventional PIR foam, however, at the same time the obtained material is easily ignitable and highly flammable. Halogen-free flame-retarded rosin-based PIR foam nanocomposites might be prepare via a two-step procedure. First, a different organically modified nanoclay (such as montmorillonite or layered double hydroxide) is dispersed in the polyol mixture, then the catalysts, silicone surfactant, halogen-free flame retardant (such as expandable graphite and diethyl ethylphosphonate), and blowing agents are added into the polyol mixture. The intergallery distance in layered double hydroxides are increased through modification with organic anions, and the layers become more organophilic. Modified LDHs become suitable for use as nanofillers, monomer, or polymer molecules can more easily penetrate between the layers. In the cited studies, layered double hydroxides were obtained in a form of Co-Al-CO_3_ LDH hexagonal platelets of 4 μm in lateral size using the urea method. Performed tests revealed that, compared to neat PIR, nanocomposite containing 45 wt.% of fire retardant and 2 wt.% montmorillonite or 3 wt.% layered double hydroxide exhibit significantly improved flame retardancy and slightly enhanced mechanical properties. Moreover, it was found that organically modified layered double hydroxide, due to promoting the formation of reinforced char layer, providing an effective barrier against heat, exhibit synergistic effect with flame retardants on improving the flame retardancy and fire behavior of rosin-based rigid PIR foam (char residue = 18.7% compare to 14% for neat PIR).

The principals of the ‘green chemistry’ are also applied for the epoxy resins, which most often are made from diglycidyl ether and bisphenol A (DGEBA) of petrochemical basis. The synthesis of more environmentally friendly epoxy resins might be performed either by the introduction of bio-based monomer or bio-based curing agent next to the petroleum derived compounds. However, these novel materials, alike petroleum-based epoxies suffer from high flammability. Enhancing the flame resistance of bio-based epoxy resins might be performed along with the process of their synthesis, via the monomer (Table 3) or curing agent.

**Figure 13:**
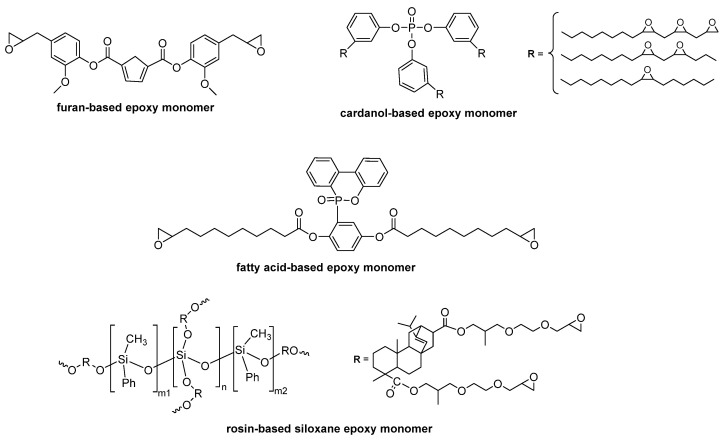
Examples of natural-based monomers used for the synthesis of bio-based materials with improved flammability.

Bio-based epoxy monomers are derived from various renewable resources, including fatty acids, eugenol, vanilin, itaconic acid, etc.; their modification (Figure 13) might result in the improved fire performance of the cured compound. 

**Figure 14:**
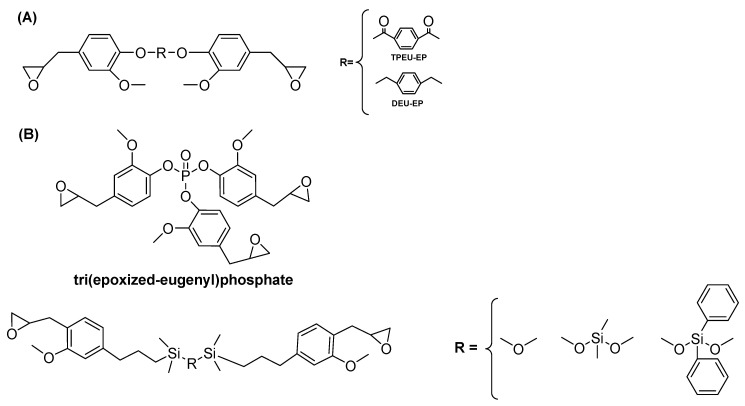
The structure of eugenol-based monomers with flame retardant properties. (**A**) obtained by linking together two eugenol molecules, followed by the epoxidation of terminal groups and curing with 4,4′-diaminodiphenylmethane or 3,3’-diaminodiohenyl sulfone; (**B**) the additional incorporation of phosphorous or silicone within the structure of eugenol.

The example of such epoxy resin is material obtained by linking together two eugenol molecules, followed by the epoxidation of terminal groups and cured with 4,4′-diaminodiphenylmethane (DEU-EP/DDM) or 3,3′-diaminodiohenyl sulfone (TPEU-EP/33DDS) (Figure 14A).

A synthesized bio-epoxy material is characterized by very high bio-based content, enhanced mechanical properties, and high-temperature charring and flammability resistance; in the case of: (1) DEU-EP/DDM—twice as large, as for DGEBA/DDM, system char residue, and lower then DGEBA/DDM-pHHR (201 kW/m^2^) and THR (16.3 kJ/m^2^) [136], and (2) TPEU-EP/33DDS—higher LOI (26,8% compared with 23.5% for DGEBA/33DDS), decreased by 68% and 40% value of pHRR and THR, compared to those of DGEBA/33DDS [137]. It is worth mentioning that by the additional incorporation of phosphorous (tri(epoxized-eugenyl)phosphate) [138] or silicone [139] within the structure of eugenol (Figure 14B), cured bio-materials exhibit higher char yield than petrochemical epoxy resins.

**Figure 15:**
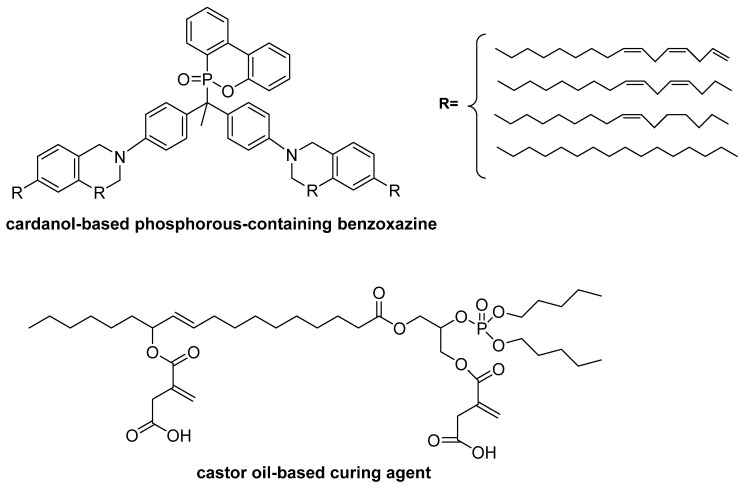
The chemical structure of bio-based curing agents applied for the synthesis of bio-based materials with improved flammability.

The bio-based character, as well as improved fire resistance of obtained epoxy materials, can be achieved throughout the introduction of a specific curing agent (Figure 15 and Table 3). 

**Figure 16:**
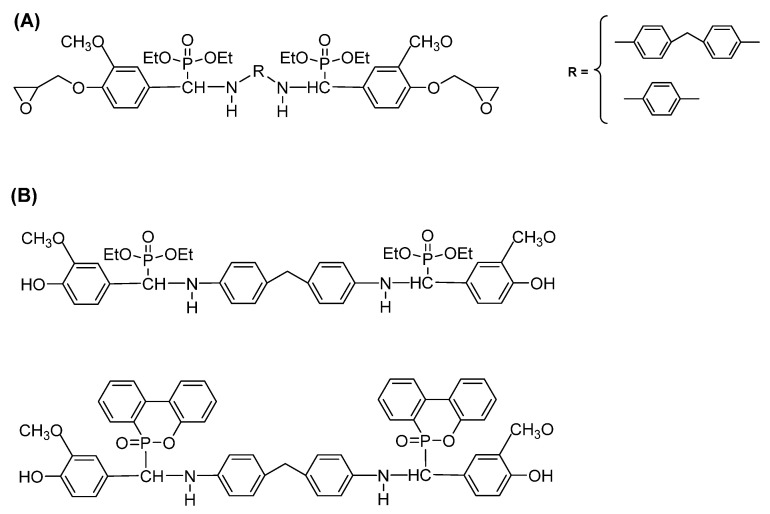
Vanilin-based monomers (**A**) and curing agents enhancing the flame retardancy of epoxy resins (**B**).

It is worth pointing out here that the same natural source might be used either for the synthesis of monomer or curing agent enhancing the fire performance of bio-based epoxy resins. The example of such a raw material is vanilin [140,141] or cardanol [142,143,144]. The introduction of vanilin-based monomer (Figure 16A) leads to a material being obtained that is characterized by excellent flame retardancy, compared to the cured petroleum-based epoxy resin (LOI = 31.3, UL-94 V-0 classification), which results from the ability of intumescent and dense char formation. On the other side, vanilin might be used for the synthesis of vanilin-based co-flame retardant with ammonium polyphosphate (Figure 16B), applied for DGEBA/DDM thermosets, which exhibit UL-94 V-0 classification and the LOI value around 29%.

Bio-polymers constitute a specific group of polymer materials, both in terms of their preparation, as well as their applications and properties. As shown above, it is possible to reduce the flammability of these important materials (including coating and construction materials) using strategies and compounds, which so far have been applied for polymeric materials, obtained from oil and gas, as well as through new solutions dedicated to specific bio-polymers.

## 6. Conclusions 

An intensive development of knowledge about polymer materials has been observed for over a century, with time, these materials have become extremely important in industry and irreplaceable in human beings’ everyday lives. However, among both scientists and government representatives around the world, much concern has been aroused due to the need to reduce as much as possible the risk of loss of possessions, health and life, resulting from the threat of the flammability of polymer materials. The application of flame retardants in order to reduce combustibility and suppress the smoke or toxic fume production from the polymers after ignition becomes particularly important. As such, currently, material research aims to achieve the best functional properties of polymer materials and environmentally friendly technologies of their synthesis and further applications. In the presented manuscript, apart from the overview of the most commonly used techniques of different approaches towards the increase of the fire resistance of polymeric materials, we have pointed out a broad range of the most recent studies related to the development of the flame retardation of bio-composite materials. The flammability of bio-composites still remains a very complex scientific problem. Unfortunately, thus far, due to the extensive diversification of available polymer matrices and additives, no single solution has been be found. Regardless of the raw materials source and the method of preparation, the polymer matrix in bio-based composites is more or less flammable, which means that for many applications of these materials it is necessary to apply different strategies to reduce their flammability. Unfortunately, a great number of studies in the field of flame retardants for bio-composites, published mostly in last couple of years, show that it is impossible to select one method or flame retardant not only for bio-based composites, but even for the particular polymer matrix. There are only varieties of methods, ways of the introduction of flame retardants and modes of their action, measures, and even parameters describing the flammability and parameters regarding the properties of flame-retardant materials. The selection of an efficient and effective strategy, and in the case of the application of flame retardants, the choice of a suitable compound requires the knowledge of the chemical mechanism and kinetics of the combustion process of a given bio-based composite, as well as related mass and heat transport processes. Numerous publications concerning the flammability of polymer materials, especially based on natural resources, prove how important and multifaceted this topic is. However, it seems necessary to deepen the studies even further by, e.g., comparing the non-flammability efficiency of the matrix with the flame retardant efficiency of applied fibers in the context of flame retardation of the composite as a whole. Nevertheless, in the case of many polymers, used as matrices in composites, well-tested and used for several years flame retardants have been applied. However, new challenges are emerging, such as the need to reduce flammability of crops-based fillers. This is why new strategies and new systems are being developed for the effective flame retardation of bio-based composites, adapted to the specificity of these materials.

## Figures and Tables

**Table 1:** Halogen-containing flame retardants.

Halogen FR	Name	Structure	LOI (%)	Ref.
Bromine-containing flame retardants	Hexabromocyclododecane (HBCD)	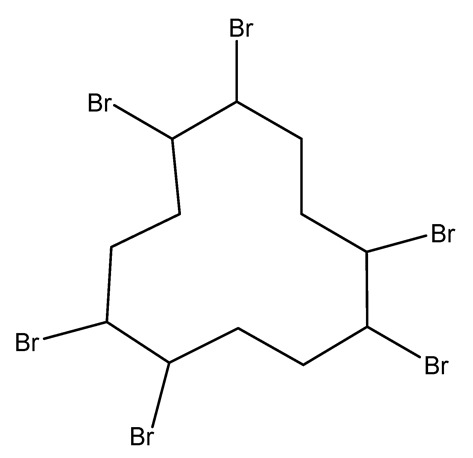	20.2 —MDI/ BDO/HTPB composite 28.3 —PS680/HCFC/HFC blends	[21,22,23,24,25,26]
	Tetrabromophthalic anhydride (TBPA)	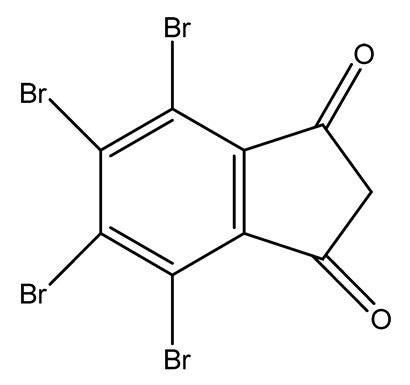	31.5 —wool/malonic acid	[27]
	Polybrominated diphenyl ether (PBDE)	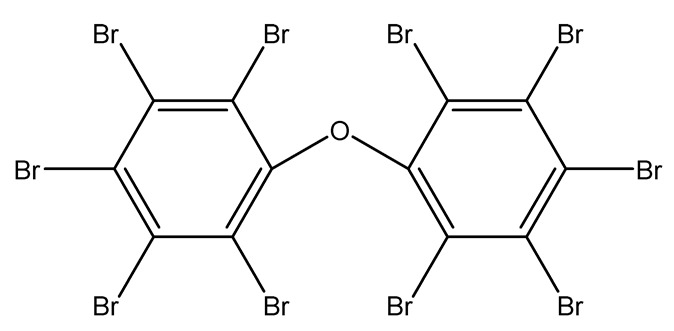	26.7 —tin(II)tungstates/PS	[28,29,30]
	Tetrabromobisphenol-A (TBBPA)	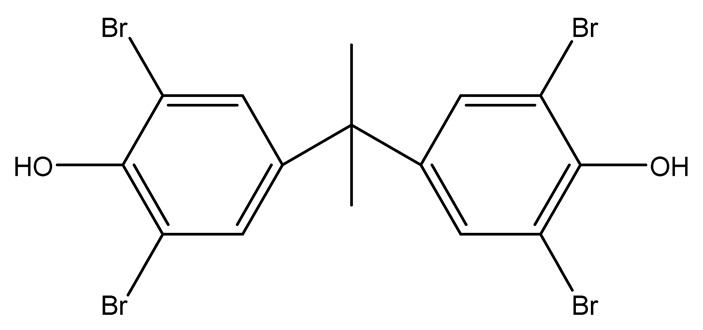	45.0 —epoxy resin/5 wt.% MMT nanoclay	[31,32,33,34]
Chlorine-containing flame retardants	1,4-di(2-hydroxyethoxy)-2,3,5,6-tetrachlorobenzene (TCHQD)	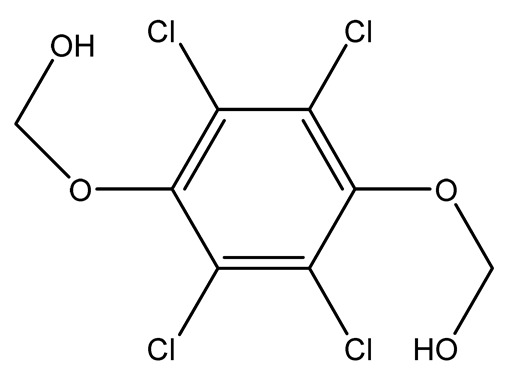	25.0 —unsaturated polyesters	[35,36]
	1,4-di(ethoxycarbonyl-methoxy)-2,3,5,6-tetrachlorobenzene (TCHQA)	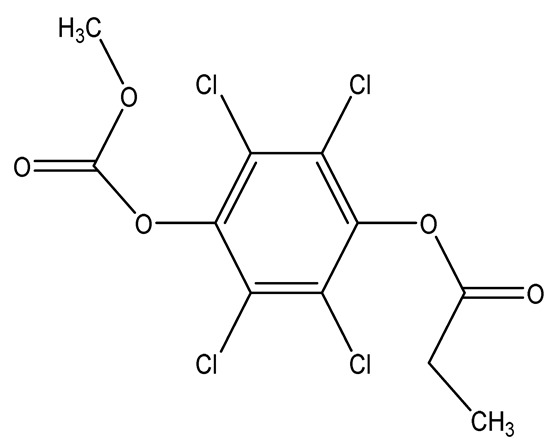	27.0 —unsaturated polyesters	[35,36]

**Table 2:** PLA composites with various flame retardants.

Composite	Remarks	Ref.
PLA/kenaf fibers/recycled carbon with a cashew nut shell liquid	- cardanol improved the thermal stability of kenaf; - the thermal stability of final composite was additionally improved by hybridization with recycled carbon (the flammability UL 90 HB test determines the flame retardancy property of all specimens)	[73]
PLA/kenaf fibers/phosphorus-based non-halogenated flame retardant (NP-100)	Addition of NP-100 flame retardant filler into the PLLA-PLA microsphere/KF composites: - improved the flammability; - decreased the flexural strength and modulus of the composites (NP-100 affects to the presence of void in the microstructure of composite).	[74]
PLA/wood fiber/mesoporous nickel phosphate	Ni-PO (when 5 wt.% ammonium polyphosphate was substituted by nickel phosphate) effects: - the reduction of the total smoke release amount of Wood Fiber-PLA composite by 43%; - gradually improved mechanical properties with the increasing loading amount of Ni-PO.	[75]
PLA/hemp/sepiolite nanoclay/ multiwalled nanotubes	Combining the flame retarding potential of carbon nanotubes and nanoclay resulted in enhanced flame retardancy of composite (ternary nanocomposite based on sepiolite and MWNTs—58% drop in pHRR, introduction of non-woven hemp fibre—45% reduction in pHRR, and 25% reduction in pHRR upon the introduction of hemp fiber into the PLA nanocomposite system; pHRR_PLA_ = 485 kW/m^2^, pHRR_PLA with hemp_ = 361 kW/m^2^, pHRR_PLA ternary nanocomposite_ = 265 kW/m^2^ and pHRR_PLA ternary nanocomposite with hemp_ = 340 kW/m^2^, respectively).	[76]
PLA/starch/ microencapsulated ammonium polyphosphate	- Microencapsulated ammonium polyphosphate within the PLA/starch biocomposites improves flame retardancy of composite and restrain the reaction between ammonium polyphosphate and starch during processing of PLA (composite PLA/starch/ microencapsulated ammonium polyphosphate: pHRR = 97 W/g; THR = 6.8 kJ/g and max HRR temp. = 365 °C compared to: 398 W/g; 13.9 kJ/g and 375 °C obtained for pure PLA). - Composites containing 30% microencapsulated ammonium polyphosphate reach UL-94 V0 with a LOI value of 41.0. The incorporation of IFR into PLA decreased the pHRR and THR of the composites.	[77]
PLA/ammonium polyphosphate (APP) microencapsulated/ polysiloxane/polyborosiloxane	The microencapsulation of ammonium polyphosphate resulted in: - improved compatibility of APP with PLA, resulting in better mechanical properties, enhanced flame retardancy (PLA composite containing 5%APP: pHHR = 512 kW/m^2^; time to ignition = 35 s and THR = 66 MJ/m^2^ compared to: 556; 39 and 78 obtained for pure PLA, respectively) and improved water resistance of the composite; - composite with BSi-APP exhibit the best flame retardancy (pHHR = 458 kW/m^2^; time to ignition = 33 s and THR = 57 MJ/m^2^ resulting from the formation of Si-O-C, Si-O-B, and B-O-P in the enhanced char residue) among the three tested FRs (APP, Si-APP and BSi-APP) to PLA under the same loading	[78]
Ramie fibers/ammonium polyphosphate	The ammonium polyphosphate: - effectively improves flame retardancy (UL94 test and LOI) due to increased char residue at high temperature; - however, disturbs the compatibility between PLA and fibers (worse dynamic mechanical properties and mechanical properties).	[79]

**Table 3:** Examples of epoxy composites with various flame retardants.

Composite	Remarks	Ref.
Epoxy/pulverized oil palm empty fruit bunch/expandable graphite	Increasing the amount of expandable graphite fillers in the composites: - significantly improved the fire resistivity and thermal properties; - however, reduced mechanical properties.	[129]
Epoxy/palm empty fruit bunch fiber/ATH/APP hybrid system	- An addition of APP enhanced the flame-resistant properties of the composite (reduced total flame time and zero drip). - The 10 wt.% ATH and 5 wt.% APP hybrid showed the most promising flame retardancy with a self-extinguishing property as well as the lowest gross heat and greatest char residue. - In order to create an acceptable FR-based systems, ATH required a greater concentration, but at the same time increasing ATH concentration resulted in deterioration of mechanical properties.	[130]
Epoxy/bimetallic metal-organic framework (MOF)/graphene oxide (GO) nano-hybrids (MOF@GO) with intumescent fire retardants (IFR)	- EP/0.5MOF@GO-9.5IFR composite exhibited a 41% decrease in peak heat release rate, 30% decrease in total smoke production compared with EP/10IFR and self-extinction behavior in the UL-94 test due to the barrier effect of highly reinforced carbonaceous char. - The intumescent epoxy composites exhibited enhanced mechanical performances (a 11% increase in tensile strength) due to improved interaction between the fillers and matrix.	[131]
Epoxy/eiphenylimidazole spirocyclic pentaerythritol bisphosphonate (PIPC)	PIPC was prepared *via* the substitution reaction between previously synthesized intermediate SPDPC (3,9-dichloro-2,4,8,10-tetraoxa-3,9-diphosphaspiro-(5,5)undecane-3,9-dioxide) and 2-phenylimidazole. An addition of PIPC: - slightly impacted the mechanical properties of epoxy composites with a low loading - in amount of 5 wt.% resulted in LOI of 29.7% and a V-0 rating in the UL 94 Test, decrease in pHRR (by 41.15%) and THR (by 21.64%).	[132]
Epoxy/flower-like nickel phyllosilicate (Ni-PS)	Fe/Ni-PS (4.0 wt.%) resulted in: LOI of 28.9 and ability of self-extinguishing with the total burning time of 12.0 s, passing the V-1 rating in UL-94 test, reduction pHRR by 20.1% and THR by 4.7 % compared to those of pure EP.	[133]
Epoxy/fish DNA-modified clays	Contribution of DNA molecules results in improvement of thermal stability and fire resistance of epoxy-clay nanocomposites (due to formation of condensed char layers during combustion caused by the release of effective suppressant agents during the decomposition of DNA structures).	[134]
Epoxy/DOPO/organoclay	A synergistic flame retardant effect DOPO and organoclay on epoxy composites (2.0 wt.% phosphorus and 4.0 wt.% organoclay) resulted in decrease of pHRR by 40% and smoke production rate by 46% when compared to neat epoxy resin (the single use of 2.0 wt.% phosphorus decreased the pHRR only to 59% of that of neat EP resin).	[135]
